# Knowledge, Attitudes, and Practices Among the General Population During COVID-19 Outbreak in Iran: A National Cross-Sectional Online Survey

**DOI:** 10.3389/fpubh.2020.585302

**Published:** 2020-12-10

**Authors:** Edris Kakemam, Djavad Ghoddoosi-Nejad, Zahra Chegini, Khalil Momeni, Hamid Salehiniya, Soheil Hassanipour, Hosein Ameri, Morteza Arab-Zozani

**Affiliations:** ^1^Department of Health Management and Economics, School of Public Health, Tehran University of Medical Sciences, Tehran, Iran; ^2^Department of Public Health, School of Health, Birjand University of Medical Sciences, Birjand, Iran; ^3^Social Determinants of Health Research Center, Qazvin University of Medical Sciences, Qazvin, Iran; ^4^Department of Public Health, School of Health, Ilam University of Medical Sciences, Ilam, Iran; ^5^Department of Public Health, School of Health, Birjand University of Medical Sciences, Birjand, Iran; ^6^Gastrointestinal and Liver Diseases Research Center, Guilan University of Medical Sciences, Rasht, Iran; ^7^Health Policy and Management Research Center, Department of Health Services Management, School of Public Health, Shahid Sadoughi University of Medical Sciences, Yazd, Iran; ^8^Social Determinants of Health Research Center, Birjand University of Medical Sciences, Birjand, Iran

**Keywords:** knowledge, attitudes, practices, COVID-19, online survey, Iran

## Abstract

**Background:** Emerged in December 2019, coronavirus disease 2019 (COVID-19) is one of the largest pandemics ever. During the early phase, little was known about public knowledge, attitudes, and practices (KAP) relating to coronavirus disease. This study was designed to determine KAP of Iranians toward COVID-19.

**Methods:** A cross-sectional online survey was carried out in Iran from February 25 to April 25 using a self-administered questionnaire on 1,480 people. COVID-19-related KAP questions were adapted from other internationally validated questionnaires specific for infectious diseases.

**Results:** All participants were aware of COVID-19. When asked unprompted, 80% of respondents could correctly cite fever, difficulty in breathing, and cough as signs/symptoms of COVID-19. Most of our sample population knew that staying at home and isolated (95.3%) as well as constant handwashing and using disinfectants (92.5%) could prevent COVID-19. However, there were also widespread misconceptions such as the belief that COVID-19 can be transmitted by wild animals (58%) and by air (48.3%). Unprompted, self-reported actions taken to avoid COVID-19 infection included handwashing with soap and water (95.4%), avoiding crowded places (93%), cleansing hands with other disinfectants (80.), and covering mouth and nose when coughing or sneezing (76.1%). The Internet and social media (94.5%) were the main coronavirus information sources. However, the most trusted information sources on coronavirus were health and medical professionals (79.3%). The majority of participants (77.0%) wanted more information about coronavirus to be available.

**Conclusion:** Our findings suggest that people's knowledge and attitude toward COVID-19 at the time of its outbreak was at a high level.

## Introduction

Having emerged in the last months of 2019, coronavirus disease 2019 (COVID-19) spread beyond an unimaginable rate, imposing a heavy burden on worldwide healthcare systems forcing almost all countries to apply quarantine rules which were unprecedented over the last century (1, 2).

Because of the characteristics of viral diseases and their transmission ways affecting a huge number of people, COVID-19 received urgent attention globally. Characteristics such as high transmission rates, long incubation period, and global spread, infecting millions of people, resulted in the fact that the virus needed to be tackled with careful design and planning, especially as in some cases a phobia began to emerge about this new virus (3).

Signs and symptoms associated with COVID-19 include fever, dry coughs, and shortness of breath. Having all three together, patients are advised to refer to healthcare facilities immediately, so that diagnosis could be quick and effective. However, because of lack of an appropriate vaccine or cure for treating and managing these patients, the best way in current situation is paying special attention to disease prevention and breaking the transmission chain of the virus (4–6).

Prevention of the disease requires public health and social measures including personal and respiratory hygiene. This includes washing hands with soap for at least 20 s, reducing interactions with other people, quarantining people who have come into contact with anyone infected, as well as quarantining infected residents. Actions like these need social awareness from both the authorities and the population at a large scale to handle the situation quickly and safely (1, 7, 8).

To protect the public in Iran, it is vital to understand the knowledge, attitude, and practice (KAP) of people so that the authorities know which areas of KAP need enhancing (9). Therefore, having these statements in mind, it seems necessary to design and implement research activities in this area. In this regard, this study, aims to assess the KAP of society about COVID-19. The results and evidence extracted from such surveys could immediately help public health planners and other health sector authorities fight this virus most effectively.

## Materials and Methods

### Study Design

This cross-sectional survey was conducted from February 25 to April 25, to assess the public knowledge, attitudes, and practices regarding the coronavirus outbreak in Iran.

### Population and Sample Size

Convenient sampling was employed in this study. The estimation of the sample size was done by assuming a minimum disease prevalence of 15%, confidence level = 95%, and *d* (margin of error) = 0.02. The calculated sample size of this study was 1,233 participants, by design effect = 1.2 reaching a sample size of nearly 1,480 participants.

### Measures

The survey questionnaire included both prompt direct questions as well as unprompted open-ended questions that allowed for multiple responses. Coronavirus KAP domains in the questionnaire included sources of information, knowledge about coronavirus, behavioral intentions, prevention practices, and attitudes toward survivors. The indicators used to assess coronavirus KAP were informed by lessons learned from similar KAP studies on other communicable diseases, especially MERS, SARS, HIV/AIDS, and Ebola (10–13). Before the final survey was completed, changes were made as required to enable a better understanding of the questions by the participants, and the arrangement of the questions was looked into to ensure its efficiency. A translate re-translate approach was used for increasing the validity of the questionnaire. Also, the CVR (73%) and CVI (81%) were calculated for the questionnaire with a sample of 10 experts.

### Data Collection

Due to maintaining social distance, the survey was done online in all provinces of the country. The questionnaire was designed on Porsline (similar to SurveyMonkey) shared on social media such as Telegram, WhatsApp, LinkedIn, and Facebook. We also tried to distribute this questionnaire on social media channels related to all provinces and cities to increase the response rate. People who were of Iranian nationality, aged 10 years or more, and agreed to participate in the survey were directed to complete the questionnaire via clicking the link (https://survey.porsline.ir/#/). The survey started with a sentence that “completing the questionnaire by the participants is considered voluntary participation.” After confirmation that participants understood this, people were guided to complete the self-report questionnaire. On average, questionnaires took ~10–15 min to complete. The ethics committee of Birjand University of Medical Sciences (BUMS) approved this study before starting the formal survey (ethical code: IR.BUMS.REC.1398.391).

### Data Analysis

Once data were collected, all questionnaires were then entered into a customized Excel-based system. All data were subsequently imported into and analyzed via SPSS v. 23.0 (SPSS Inc., Chicago, Illinois, USA). Descriptive statistics were then generated for national-level estimates (proportions) and their 95% CIs.

## Results

A total of 1,480 people responded to the questionnaire, yielding a response rate of 70.2%. This response rate is based on the number of views by participants and the number that fulfilled the survey. The mean age of participants was 31.29 years. Among the respondents, 797 (57.2%) were female. Moreover, 782 (53.1%) were married. Of all respondents, 41.9% were between the ages of 20–31 years. Also, above 80% of respondents hold degrees of higher education (Table 1).

**Table 1:** Sociodemographic characteristics of respondents (*n* = 1,480).

**Characteristic**		***N***	**Percent^**+**^**
Sex^*^	Male	597	42.8
	Female	797	57.2
Age^†^	≤20	150	10.3
	21–30	612	41.9
	31–40	461	31.6
	41–50	166	11.4
	>50	70	4.8
Marital status^‡^	Single	692	46.9
	Married	782	53.1
Educational level^§^	Lower diploma	51	3.5
	Diploma	200	13.6
	Associate degree	115	7.8
	Bachelors	595	40.3
	Master	395	26.8
	PhD	120	8.0
Region^**^	South	144	9.8
	North	412	28.0
	West	411	27.9
	East	288	19.6
	Center	217	14.7

* *Missing values for sex = 86*.

† *Missing values for age = 21*.

‡ Missing values for marital status = 6.

§ *Missing values for education = 4*.

** *Missing values for region = 8*.

+ *Valid percent*.

### Awareness and Risk Perception

The majority of respondents (84.5%) had heard of coronavirus disease 2019 (COVID-19) before the interview. Overall, 84.5% of respondents were aware that it is possible to survive and recover from COVID-19. Approximately 60% of respondents (59.6%) perceived themselves to be at some risk of contracting COVID-19.

### Knowledge of Coronavirus Cause, Transmission, Signs, and Symptoms

In an open-ended question, the most common perceived cause/origin of coronavirus was “virus” (94.4%); only 31.7% linked coronavirus to a “bats, monkeys, and wild animals.” Very few respondents mentioned that coronavirus is caused by “Bacteria” (6.7%), “Evildoing/Sin” (4.1%), or “Parasites” (3.7%).

In response to an unprompted open-ended question, the most frequently cited modes of transmission were shaking hands with an infected person (91.9%), kissing and hugging (90.1%), and being in contact with the saliva of an infected person (87.2%) (Table 2).

**Table 2:** Coronavirus-related awareness, risk perceptions, and knowledge (*n* = 1,480).

**Indicator**		**Percent**	**95% CI**
Awareness and risk perception	Heard of coronavirus	84.5	82.6–86.34
	Expressed that coronavirus existed in Iran	98.1	97.4–98.8
	One of your family or relatives of friends infected by coronavirus	84.5	82.6–86.3
	Aware of possibility to have coronavirus without showing signs/symptoms	79.5	77.4–81.5
	Aware of possibility to survive and recover from coronavirus	84.5	82.6–86.3
	Aware of coronavirus call center to report sick persons and deaths	52.8	50.2–55.3
	Perceived some risk of contracting coronavirus	59.6	57.1–62.1
Knowledge of coronavirus cause	Virus	94.4	93.2–95.5
	Bats/cats/other wild animals	31.7	29.3–34.1
	Parasites	3.7	2.7–4.6
	Bacteria	6.7	5.4–7.9
	Evildoing/sin	4.1	3.1–5.1
	I don't know/ not sure	5.5	4.3–6.6
Knowledge of coronavirus modes of transmission	Air	44.6	42.1–47.1
	Eating “animal meat”	31.9	29.5–34.2
	Eating infected fruits and vegetable	24.2	22.1–26.3
	Saliva of an infected person	87.2	85.5–88.9
	Urine and feces of an infected person	37.3	34.8–39.7
	Breast milk of an infected person	18.1	16.1–20.1
	Shaking hands with an infected person	91.9	90.5–93.3
	Kissing and hugging	90.1	88.6–91.6
	Others	27.2	24.9–29.4

**Figure 1:**
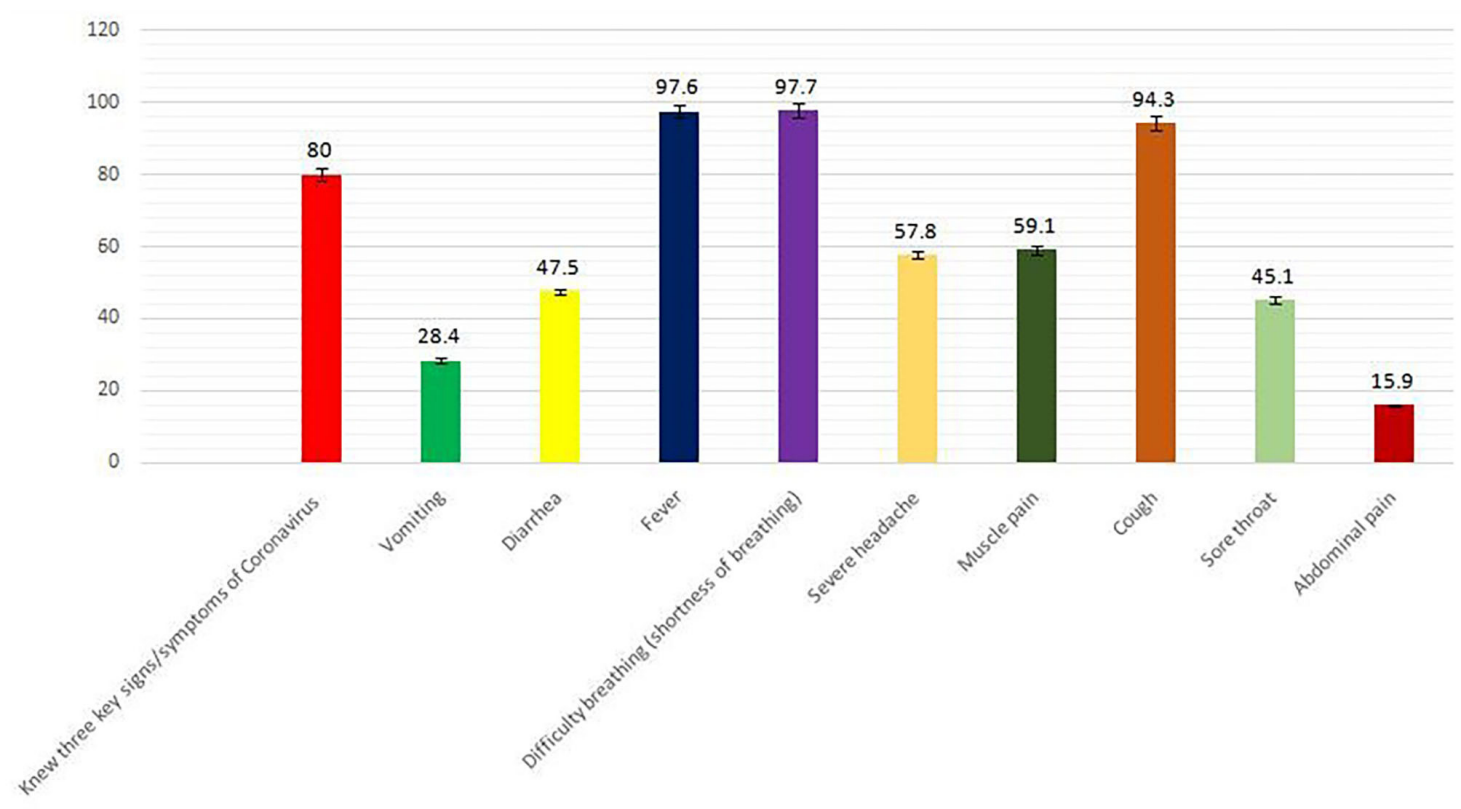
Knowledge of coronavirus signs/symptoms.

Overall, 80% of respondents could state, without prompting, three key signs/symptoms of Coronavirus: “difficulty breathing” (97.7%), “fever” (97.6), and “cough” (94.3%) (Figure 1).

### Knowledge of Coronavirus Prevention and Treatment

Knowledge of coronavirus prevention and treatment was high among respondents in that nearly everyone knew that coronavirus can be prevented by staying at home and reducing contact with people (95.3%) as well as constant hand washing and using disinfectants (92.5%).

Furthermore, 75.1% of respondents expressed that early treatment of coronavirus could increase survival and reduce the chance of transmission within the household (Table 3).

**Table 3:** Coronavirus-related knowledge, behavioral intentions, and practices.

**Indicator**		**Percent**	**95% CI**
Knowledge of coronavirus prevention and treatment	Coronavirus can be prevented by avoiding contact with blood and body fluids	39.8	37.3–42.3
	Coronavirus can be prevented by not touching anyone else	84.3	82.4–86.1
	Early treatment increases chance of surviving coronavirus	75.1	72.9–77.3
	Early treatment reduces further coronavirus spread within household	72.8	70.5–75.1
	Coronavirus can be prevented by regular washing hands with soap and water, and also using disinfectants	92.5	91.1–93.8
	Coronavirus can be prevented by stay at home and maintaining social distance	95.3	94.2–96.3
	Coronavirus can be prevented by using a face mask	69.8	67.4–72.1
Misconceptions of coronavirus transmission, prevention and treatment	Coronavirus can be prevented by bathing with salt and hot water	47.6	45.1–50.1
	Coronavirus can be transmitted through the air	48.3	45.7–50.8
	Coronavirus can transmitted by wild animals	58.0	55.5–60.5
	Coronavirus can be treated successfully by traditional healers	15.1	13.2–16.9
	Coronavirus can be treated successfully by spiritual healers	4.2	3.1–5.2
Perceptions of health facilities	Health facility will take care of sick person	76.8	74.6–78.9
	Health facility will definitely cure sick person from coronavirus	74.5	72.3–76.7
	Health facility will not be able to do anything for sick person	12.0	10.3–13.6
Perceptions of coronavirus treatment center and quarantine measures	Persons diagnosed with coronavirus must be admitted in coronavirus treatment center	86.7	84.5–88.4
	Direct contacts of patient diagnosed with coronavirus must be quarantined for 2 weeks	94.7	93.5–95.4
Health-seeking behavioral intentions	Would go to health facility if coronavirus suspected	65.4	62.9–67.8
	Would go to health facility if had a high fever	75.2	73.0–77.4
Behavioral intentions if family member suspected of coronavirus	Call the hospital/coronavirus hotline	54.7	52.1–57.2
	Take the person to the hospital	49.5	46.9–52.1
	Avoid all physical contact and bodily fluids of that person	74.6	72.3–76.8
	Help care for the person at home	40.1	37.6–42.6
	I do not know/not sure	4.5	3.4–5.5

### Misconceptions of Coronavirus Transmission, Prevention, and Treatment

There were widespread misconceptions about coronavirus transmission, prevention, and treatment. Nearly half (47%) of respondents said they could protect themselves from coronavirus by washing hands with salt and hot water solution and nearly half again (48.3%) said coronavirus is transmitted by air while 58% of the respondents expressed that coronavirus is transmitted by wild animals. Moreover, 15.1% of respondents perceived that traditional healers could successfully treat coronavirus (Table 3).

### Health-Seeking Behavioral Intentions and Self-Reported Prevention Practices

Most of the respondents (75.2%) reported that they would go to a health facility if experiencing a high fever. Nearly all respondents (97%) reported taking some preventive action since learning about coronavirus. Handwashing with soap and water was the most prevalent behavior reported in unprompted response (95.4%), followed by avoiding crowded places (93%), cleaning my hands with other disinfectants (80.9 %), and covering mouth and nose when coughing and sneezing (76.1%).

### Attitudes Toward Coronavirus Survivors

Only 10.6% reported that they would not welcome back a coronavirus survivor into the community through 49.5% would refuse to buy fresh vegetables from a shopkeeper who survived coronavirus.

### Perceptions of Coronavirus Vaccine and Experimental Treatment

Of respondents, 73.2% reported they would accept an approved coronavirus vaccine if it were to become available, while, only 26.1% said they would accept experimental treatments that have not been trialed (Table 4).

**Table 4:** Attitudes toward coronavirus survivors and perceptions of coronavirus vaccine and experimental treatment.

**Indicator**		**Percent**	**95% CI**
Attitudes toward coronavirus survivors	Student who survived coronavirus puts others in class at risk of infection	45.9	43.3–48.4
	Would not buy from shopkeeper who survived coronavirus	49.5	46.9–52.1
	Would not welcome coronavirus survivor into community	10.6	9.1–12.1
Perceptions of coronavirus vaccine and experimental treatment	Would accept an approved coronavirus vaccine for self	73.2	70.9–75.4
	Would accept an approved coronavirus vaccine for children	73.8	71.5–76.1
	Would accept experimental coronavirus treatment for self	26.1	23.8–28.3
	Would accept experimental coronavirus treatment for relative	19.1	17.1–21.1

### Sources of Receiving Coronavirus-Related Information

**Figure 2:**
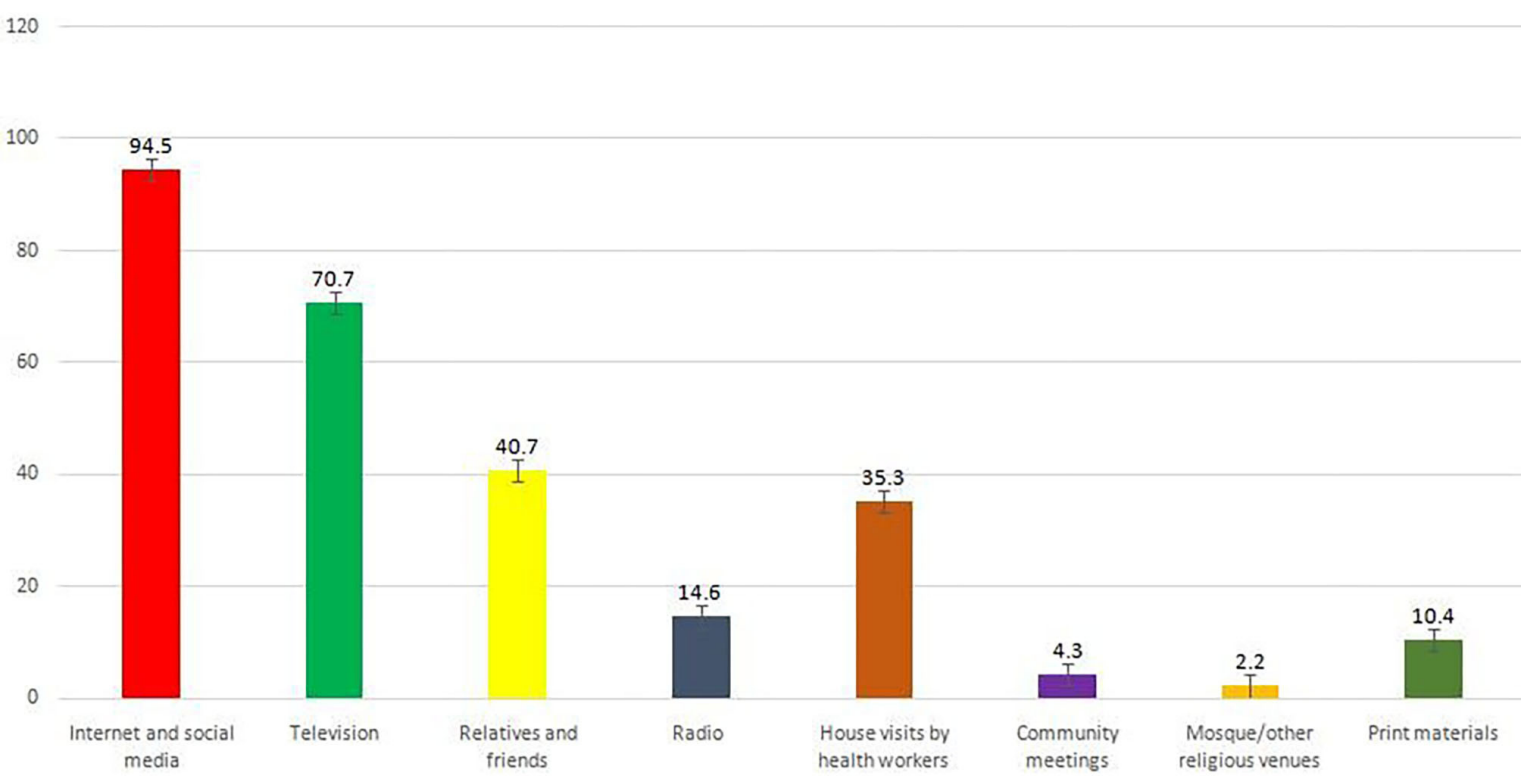
Sources of receiving coronavirus-related information.

The Internet and social media (94.5%) is the primary coronavirus information channel mentioned by respondents in an open-ended question, followed by television (70.7%), relatives and friends (40.7%), and house visits by health workers (35.3%) (Figure 2).

**Figure 3:**
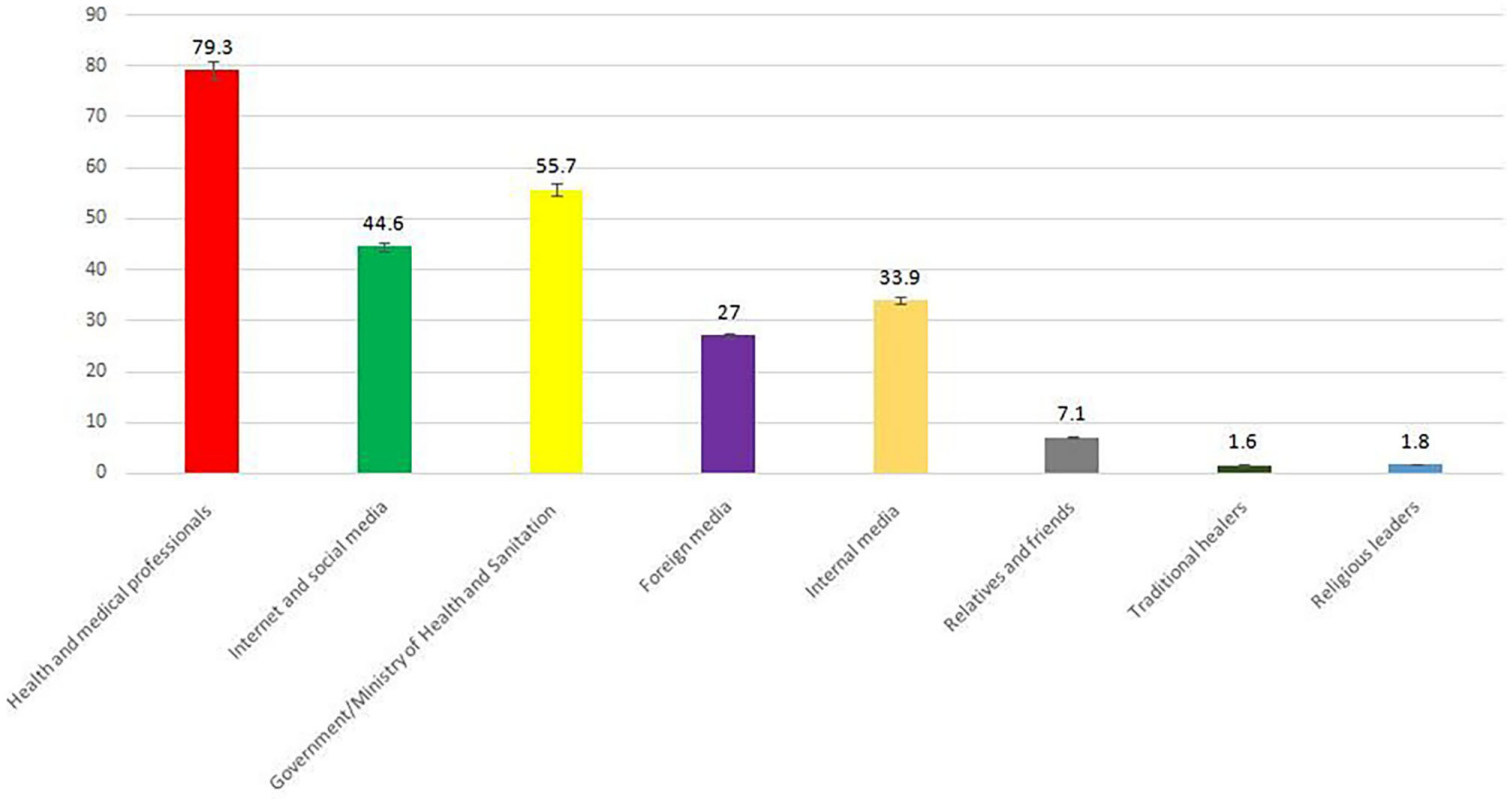
Trusted sources of coronavirus-related information.

The most trusted information sources on coronavirus were health and medical professionals (79.3%), the Government/Ministry of Health (55.7%), and the Internet and social media (44.6%) (Figure 3).

### Coronavirus Information Gaps

The majority of participants (77.0%; 95% CI, 74.8–79.1) wanted more information about coronavirus and specifically mentioned wanting to know more about providing medical care and treatment for infected people (75.5%; 95% CI, 73.3–77.7) as well as how to prevent the disease (62.2%; 95% CI, 59.7–64.6), while about half of the respondents wanted more information on coronavirus' signs/symptoms and its cause/origin (54.3%; 95% CI, 51.7–56.8 and 47.2 95% CI, 44.6–49.7, respectively).

## Discussion

This study aimed to investigate the level of KAPs of the general population around COVID-19 in Iran. In this regard, the KAP findings suggest that while during the outbreak, awareness and preventive behaviors related to COVID-19 were promoted well in Iran, misconceptions and discriminatory attitudes to those who survived coronavirus were common.

The study also highlighted the gap between the perceived susceptibility of catching the virus and reality. For example, while almost all adults were at risk of catching coronavirus, only 60% of people considered themselves at risk (14). Therefore, proper training about the susceptibility of all people and the possibility of getting the virus can increase awareness among individuals and help prevent infection. It is clear that each epidemic has its unique characteristics; hence training in various areas of the disease, including susceptibility, is essential to prevent the spread of coronavirus (15).

Knowledge about the signs and symptoms of coronavirus disease was at a high level among people with over 80% providing correct answers. This compares with studies in various countries, including China (16), USA (17), and India (18), revealing that people are highly aware of coronavirus, due to information in the mass media, including radio, television, social media, and official authorities' efforts like the Ministry of Health programs.

Moreover, according to the results, 95% of people have stated that staying at home and obeying health protocols will prevent them from being infected. In this regard, studies have also illustrated that staying at home and maintaining social distance has a positive effect on reducing disease transmission (16). Countries that did not enforce traffic regulations or imposed them later are suffering from a higher prevalence (19). To maintain social distancing, several measures have been taken into account in Iran, including the prohibition of commuting on intercity routes, avoiding crowded places, and discontinuation of high-risk jobs. However, despite the numerous warnings of the government and the Ministry of Health, and also owing to the lack of effective laws, some people did not follow these measures.

In line with this, some misconceptions about the disease persisted. For instance, 47% of people believe the virus is killed by the saline solution, and 58% believe that the virus is transmitted through wild animals (20). Any misconceptions about the disease and ways to prevent it could lead to a drastic increase in the incidence rate. Therefore, more detailed comprehensive training and information may need to be disseminated through the media, health practitioners, researchers, and other stakeholders.

However, positive key findings show that 75% of people stated they would go to a health facility if they had a fever, and 90% of people say they have taken preventive measures since the onset of coronavirus such as washing their hands with soap and water and avoiding crowded areas.

One area of concern revealed by the study is the stigma of coronavirus survivors. Some 10% of people said they would not welcome a survivor of coronavirus into the community while 49.5% stated they would refuse to buy fresh vegetables from shopkeepers who had survived coronavirus. This may raise main concerns about the stigma attached to patients who have the disease among their community. Therefore, measures should be taken to increase awareness at the community level to reduce stigma (18, 21).

From the publicly available information sources, the most important source for acquiring knowledge for most people is social media. Therefore, it is necessary to provide accurate, precise, and timely information to the public (22, 23). Of course, the production and presentation of appropriate content through virtual networks should be under the supervision of relevant organizations such as the Ministry of Health. Also, those in charge of training in virtual networks should be identified so that they can be held accountable in case of discrepancies between the trainings and the correct evidence.

Finally, more than 50% of people stated a need for more information and knowledge about the treatment and diagnosis of the virus. Since this is a new epidemic, it is important to have reliable and up-to-date information on all aspects of the disease; from prevention to diagnosis and treatment to acquire coherence and cohesion in people's behavior using scientific protocols, not gossip or fake news in social media and on the Internet.

### Strength and Limitation

This study is the first to be conducted nationally in Iran about the KAPs of the general population regarding coronavirus and has a high sample size. Since the study was conducted online only and people who have access to social media networks have been included in the study, it would not be prudent to generalize the results to the whole community (people without smartphones and less literate).

## Conclusion

In conclusion, people's knowledge, attitudes, and performances about the disease are at a high level, but there are misconceptions about the disease. Also, the fear of dealing with recovered patients and the lack of communication and knowledge around recovered patients are at a high level. However, the disease is unlikely to be transmitted to a person after recovery, so training and information via the media can help reduce misconceptions and the stigma around recovered patients in the community.

## Data Availability Statement

The raw data supporting the conclusions of this article will be made available by the authors, without undue reservation.

## Ethics Statement

The studies involving human participants were reviewed and approved by Birjand University of Medical Sciences, Ethical Committee (Ethical code: IR.BUMS.REC.1398.391). Written informed consent to participate in this study was provided by the participants' legal guardian/next of kin.

## Author Contributions

MA-Z and EK: idea, design, and editing. MA-Z, EK, DG-N, KM, and ZC: preparing the questionnaire. KM, ZC, HA, SH, and HS: data collection. SH, EK, and HS: analyses. All authors: writing draft and approval of final draft.

## Conflict of Interest

The authors declare that the research was conducted in the absence of any commercial or financial relationships that could be construed as a potential conflict of interest.
